# Glasses Called “London Smoke” Are Better for Shielding the Eyes from Too Intense Light

**Published:** 1874-01

**Authors:** 


ART. III.—*Glasses, called "London Smoke," are preferable to blue glasses for shielding the eyes from too intense light.*

**Translated from Annales D'Oculistique, of Sept. and Oct., 1873, by FRANK W. ABBOTT, M. D.**

The Oculist is often called upon to protect the eyes of his patients against the action of too intense light; so also, among the invalids who frequent ophthalmological clinics, we see a great number who wear glasses designed to temper the light. The only difference which is noticed among them is, that some employ for this purpose blue glasses, while others prefer those of a gray color, called "smoked."

Blue glasses are those which a majority of Oculists especially recommend, but if they should be asked the reasons for their predilection, they would certainly find it difficult to give them, for lack of a scientific basis upon which they could rest. The examination of this question which I have made the subject of special attention, has led me to results which I will briefly state.

To give a clear idea of the problem before us let us analyize it from the point of view of the Young—Helmholtz theory, (*Vide*

Buffalo Medical and Surgical Journal, May, 1872, pp. 376 *et seq.*, TRANS.), not only because this best unites and explains the facts of dissimilar nature which belong to the domain of the perception of light, and of the colors, but especially because some facts, recently learned, (1) give to this theory a solid, and, so to speak, anatomical basis. Among these fact we should mention in the first place the discovery of Mueller of the fatty and colored drops which are found in the retina of birds and reptiles, drops which only allow the passage of rays of light which are very nearly of the same nature, whilst they hinder all the others.

Our right to admit the existence, in the retina of man, of an apparatus receiving the three primary colors is founded: 1st, upon the cases of congenital color-blindness, in which the eye is incapable of perceiving one of the primitive colors; 2d, upon the researches of Leber (2) who has demonstrated the existence of color-blindness following atrophy of the optic nerve and some other amblyopias. Such patients may be blind to red, and perhaps also to green, but they are never blind to blue; 3d, upon the researches of Dr. Weinow, (3) who has found that the periphery of the visual field loses not only receptivity for red, as Schelske had already proved, (4) but also for blue and violet, in such a manner that, towards the extremity of the periphery, there is found a zone possessing elements adapted only to receive the single color green.

From what precedes we can draw only one conclusion, that is, in order to protect the eyes against too intense light, it is necessary to employ glasses, which abate equally all the colors of which sunlight is composed.

If we place before the slit of a solar spectroscope first a blue glass, and then a glass of smoke color, in such a way that the luminous rays before being decomposed by the prism are tempered by these glasses, it is not difficult to recognize which of these glasses abates the solar light most equally. If the luminous rays pass through a blue glass, the colors which are the most enfeebled are the yellow and green, that is, the colors of the middle of the

---

(1) Dobrowolsky. Zur Anatomie der Retina, in Reicherts. n. Bois Reymond's Archiv., 1871.
(2) Archiv. fur Ophthalmologie, Bd. XV. Aqth., 2 S. 26–107.
(3) Id. Bd XVI., Abth. 1, S. 212–224.
(4) Id. Bd. IX, Abth 3, S. 39–49.

spectrum, whilst the red is scarcely affected and the blue and violet not at all. If the eye perceives any abatement in the bright red, near the line C, it no longer notices this abatement from B to A in the dark red color. This indicates that blue glasses, in addition to blue rays, allow a great quantity of red rays to pass, especially of the dark red, which correspond to the extremity of the spectrum. The brightest part of the spectrum, which is ordinarily found in the green color, not far from the yellow, is transported by a blue glass to the middle of the blue color.

If, on the contrary, the rays pass through smoke-colored glasses, the whole spectrum appears to the eye to be equally weakened from one extremity to the other. I certainly do not mean to say that all the colors are weakened in an absolutely equal degree, but simply that if any inequality exists in the weakening of the colors it never attains such a degree that our eyes can notice it. The impression produced upon our eyes, when we put on smoke-colored spectacles, accords in the highest degree with what has just been said: a sunny day then appears to us foggy and dark, as though there were no longer any sun's rays, and the sky were covered with thick clouds.

In this manner I have made my experiments upon the different kinds of blue and smoke colored glasses found at the shops, and I have always obtained the same results; the only difference depends on the darker or lighter tint of the glasses.

When our aim is to protect the nervous apparatus of the eye against the action of too intense light, it is naturally necessary to choose for this purpose spectacles possessing qualities adapted to defend equally the three kinds of nervous elements of the retina, by procuring for all of them repose and tranquility. Now, as we can only attain this end by using spectacles which weaken equally all the colors composing white light, we are forced to the conclusion that to protect the eye properly it is necessary to use smoke-colored glasses.

It is scarcely necessary to add, after what has just been said, that by the use of blue glasses, we resolve but very imperfectly the given problem. Blue glasses protect best nerves which are sensitive to yellow, and not at all those which are affected by blue.

In advising patients to wear blue glasses, even for working, we force just those nerve elements of the retina which are sensitive to blue, to take upon themselves all the work which was previously distributed among all the kinds of nerves of the retina. If patients, whose eyes are irritable, experience from the use of blue glasses some alleviation, some rest, it is probably because two kinds of nerves are soothed by them; but, as a consequence, afterwards, when the patients have recovered, and they try to lay aside the glasses, it is with much difficulty that they become habituated to the light of the sun, or even to the light of day, probably because all the nerves of the eye have not been equally protected. The nerves, which have been the most so, to speak accurately those which are sensitive to the yellow rays, have been unaccustomed to the influence of their ordinary excitant.

This great practical inconvenience, which is not rare in those who wear blue glasses, has already influenced some Oculists to cease prescribing them, and to prefer smoke-colored glasses.

Doctor Berthold, of Konigsberg, has told me that, in his practice he has obtained from the use of grey glasses much better results as compared with those which he formerly obtained by the use of blue glasses, and stated that after using grey glasses the eyes accustom themselves very readily to solar light.

In prescribing blue glasses, we should also not forget that the sensitiveness of the eye for colors is far from being equal; that it increases from the red to the violet, so that the sensitiveness of certain eyes for the blue and the violet is twenty times as great as for the red at the other end of the spectrum.

So that by prescribing blue glasses and protecting against the action of light, the least sensitive nerves, we impose all the work upon the nerves endowed with the greatest sensitiveness. This prescription would only be justified in cases where it was demonstrated that our eyes, while distinguished for showing a very great sensitiveness for blue, endures it longer and more easily than the others without any fatigue, but up to the present time we have no data on this subject.

